# Supplementary material for: Identification of MLH2/hPMS1 dominant mutations that prevent DNA mismatch repair function
Source: Commun Biol. 2020 Dec 10;3:751. doi: 10.1038/s42003-020-01481-4 (PMC7730388; doi:10.1038/s42003-020-01481-4)
Supplement: Supplementary file 1 — Supplementary Information [file 42003_2020_1481_MOESM1_ESM.pdf]

Supplementary Information for:

Identification of *MLH2/hPMS1* dominant mutations that prevent DNA mismatch repair function

Authors: Gloria X. Reyes, Boyu Zhao, Tobias T. Schmidt, Kerstin Gries, Matthias Kloor and Hans Hombauer

Supplementary Figures 1-7

Supplementary Tables 1-7

Supplementary References

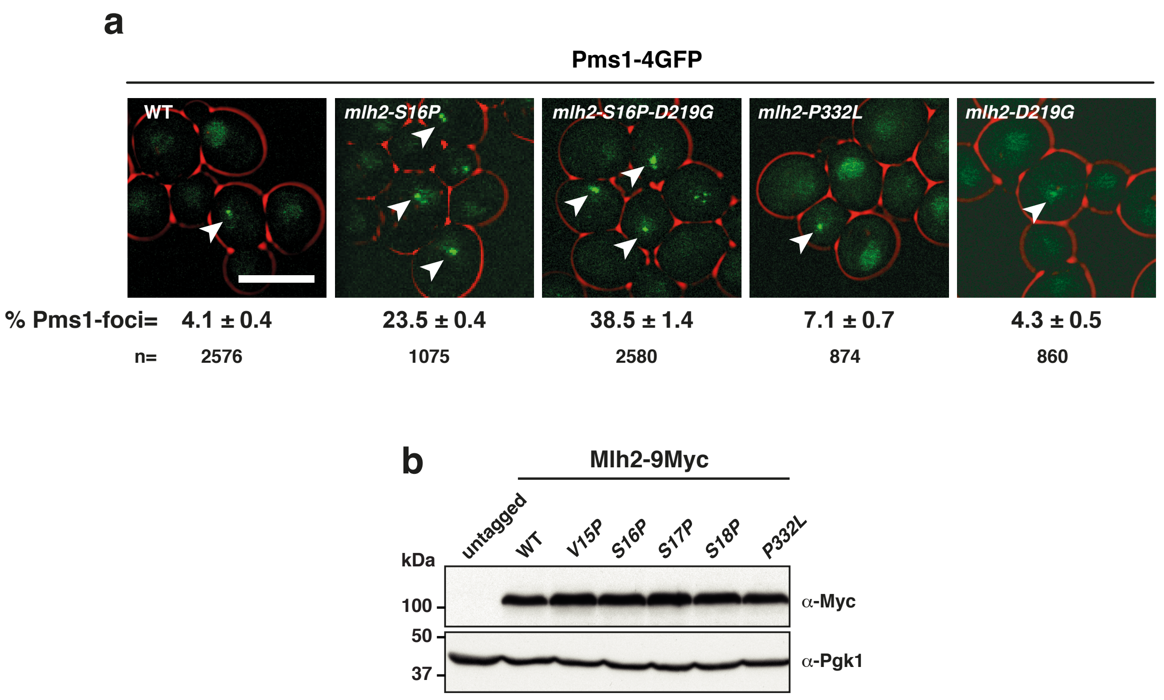

Supplementary Fig. 1. Dominant mutations in Mlh2-NTD results in accumulation of Pms1-foci. a, Representative confocal images of Pms1 foci in strains carrying *mlh2* mutant alleles. Scale bar represents 5 μm. The percentage of cells containing Pms1-4GFP foci is indicated below. Total number of imaged cells (n=) is indicated. **b**, Whole-cell lysates of logarithmically growing cells expressing WT or mutant *mlh2* alleles (tagged with a C-terminal 9xMyc) were analyzed by western blot with an anti-Myc antibody. Pgk1 was used as loading control.

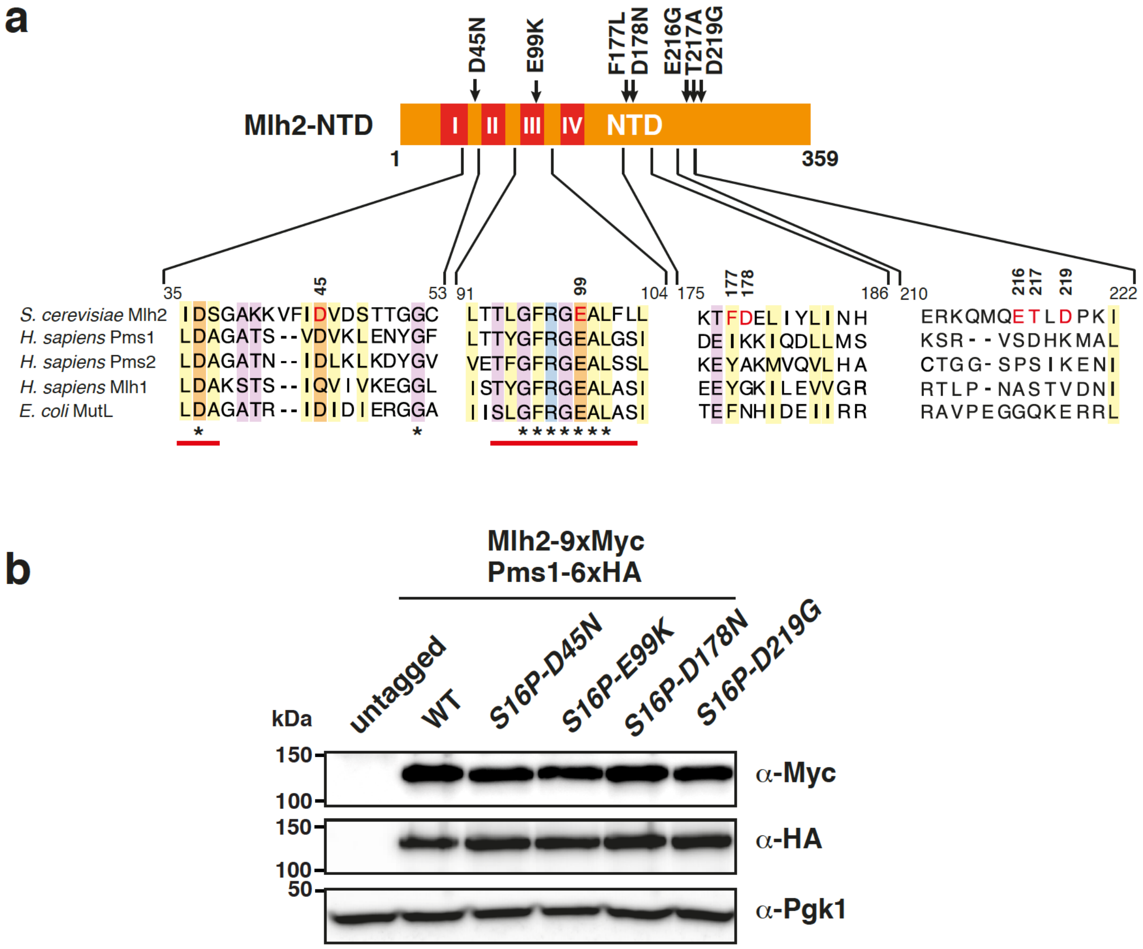

Supplementary Fig. 2. Identification and characterization of *mlh2-S16P* mutational enhancer mutations. a, Diagram of Mlh2-NTD indicating *mlh2-S16P* enhancer mutations (arrows). Boxes in red represent ATPase motifs conserved among MutL homologs. Below, protein sequence alignment of *S. cerevisiae* Mlh2, *H. sapiens* hPMS1, hPMS2 and hMLH1 and *E. coli* MutL. *Mlh2-S16P* mutational enhancer mutations are marked in red. Conserved hydrophobic residues are shaded in yellow, basic in blue, acidic in orange and others in purple. Red bars denote ATPase motifs I (partial sequence) and III. (*) indicates invariable residues across species. **b**, Mlh2 and Pms1 protein expression levels in WT and *mlh2* double mutants were determined by western blot with anti-Myc and anti-HA antibodies. *MLH2* and *PMS1* genes were tagged at the C-terminus (chromosomal locus) with 9xMyc and 6xHA, respectively. Pgk1 was used as loading control.

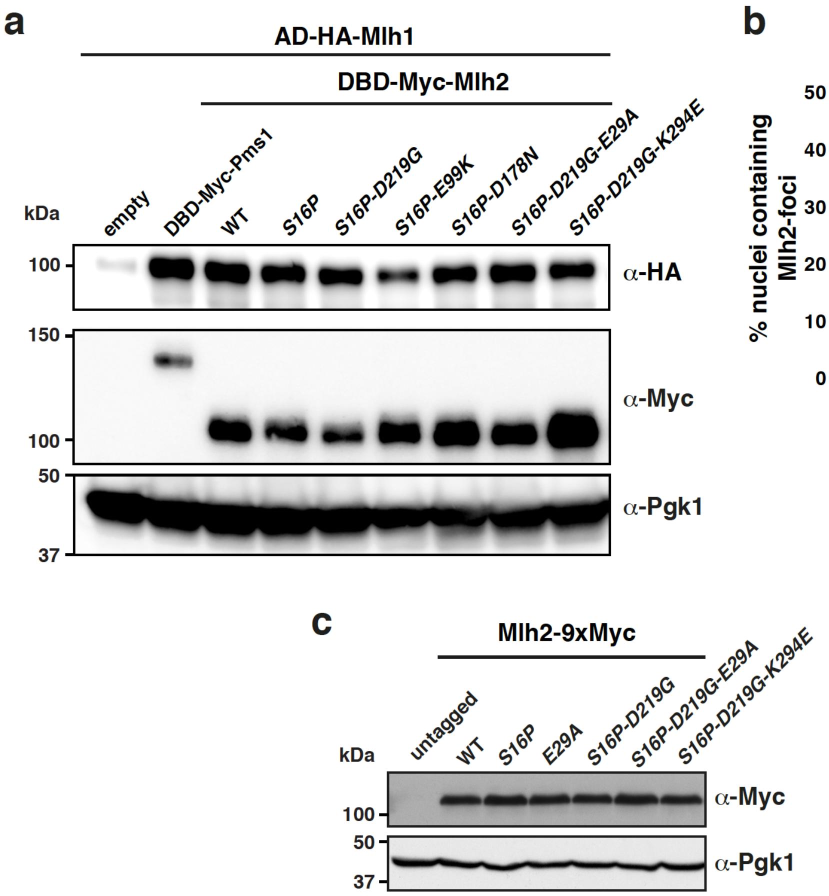

Supplementary Fig. 3. Characterization of *mlh2* double mutant alleles. a, Protein expression levels of prey (AD-HA-Mlh1) and bait proteins (DBD-Myc fused to Pms1, Mlh2 or *mlh2* mutants) used in Y2H analysis (shown in Fig. 4a) were determined by western blot with an anti-HA and anti-Myc antibodies. Pgk1 was used as loading control. AD and DBD stands for "activation domain" and "DNA binding domain", respectively. **b**, Quantification of Mlh2-4GFP foci in the indicated strains. (***) $p < 0.001$. **c**, Mlh2 expression levels in logarithmically growing WT and *mlh2* mutant strains were determined by western blot with an anti-Myc antibody. Strains were tagged at the *MLH2* locus (C-terminus) with 9xMyc. Pgk1 was used as loading control.

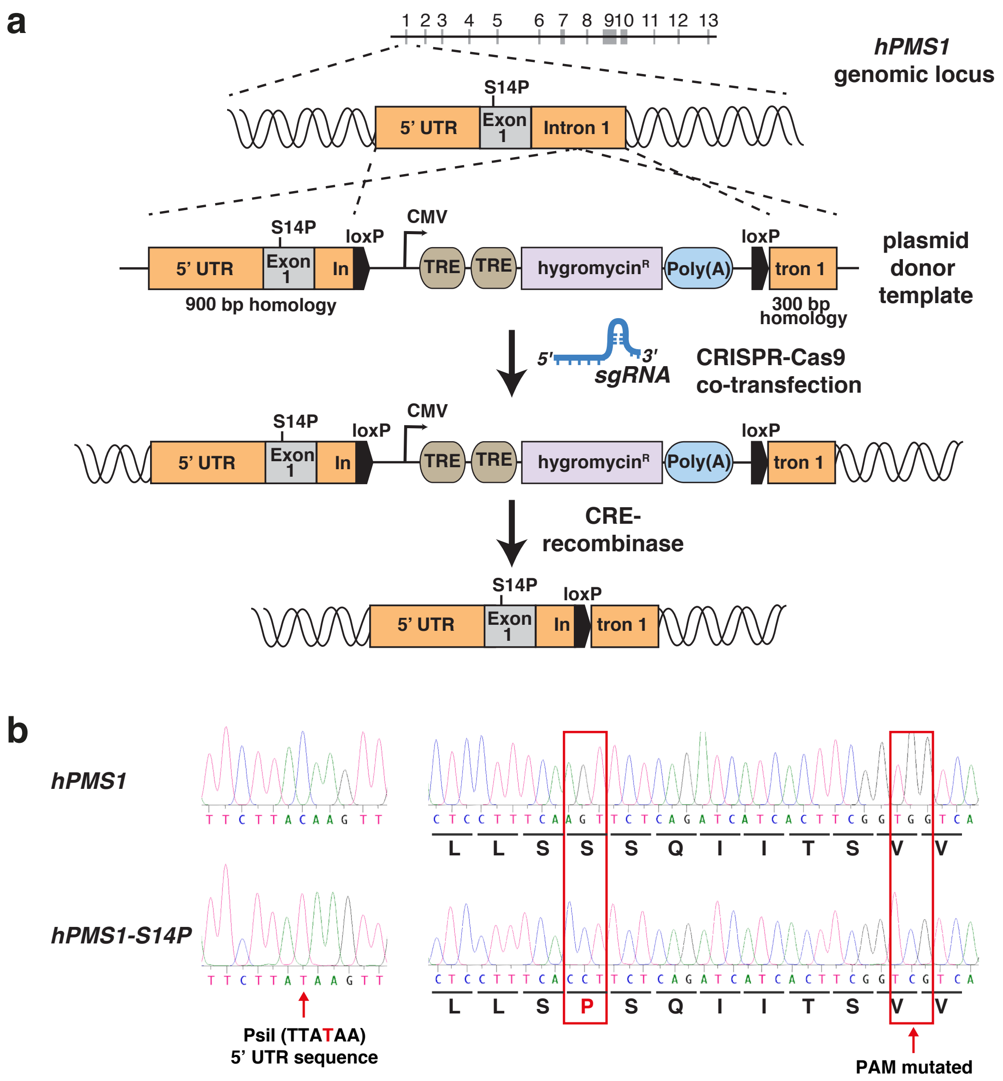

Supplementary Fig. 4. Generation of *hPMS1-S14P* HAP1 mutant cell lines. a, CRISPR-Cas9 editing strategy used to introduce the *hPMS1-S14P* point mutation in HAP1 cells. **b**, Sequencing chromatograms of two DNA regions of the *hPMS1* gene, indicating an upstream mutation (at the 5' UTR) that creates a *Psi*I site for screening purposes, the S14P mutation, and one mutation at the PAM motif that prevents Cas9-mediated cleavage after successful recombination with the donor plasmid.

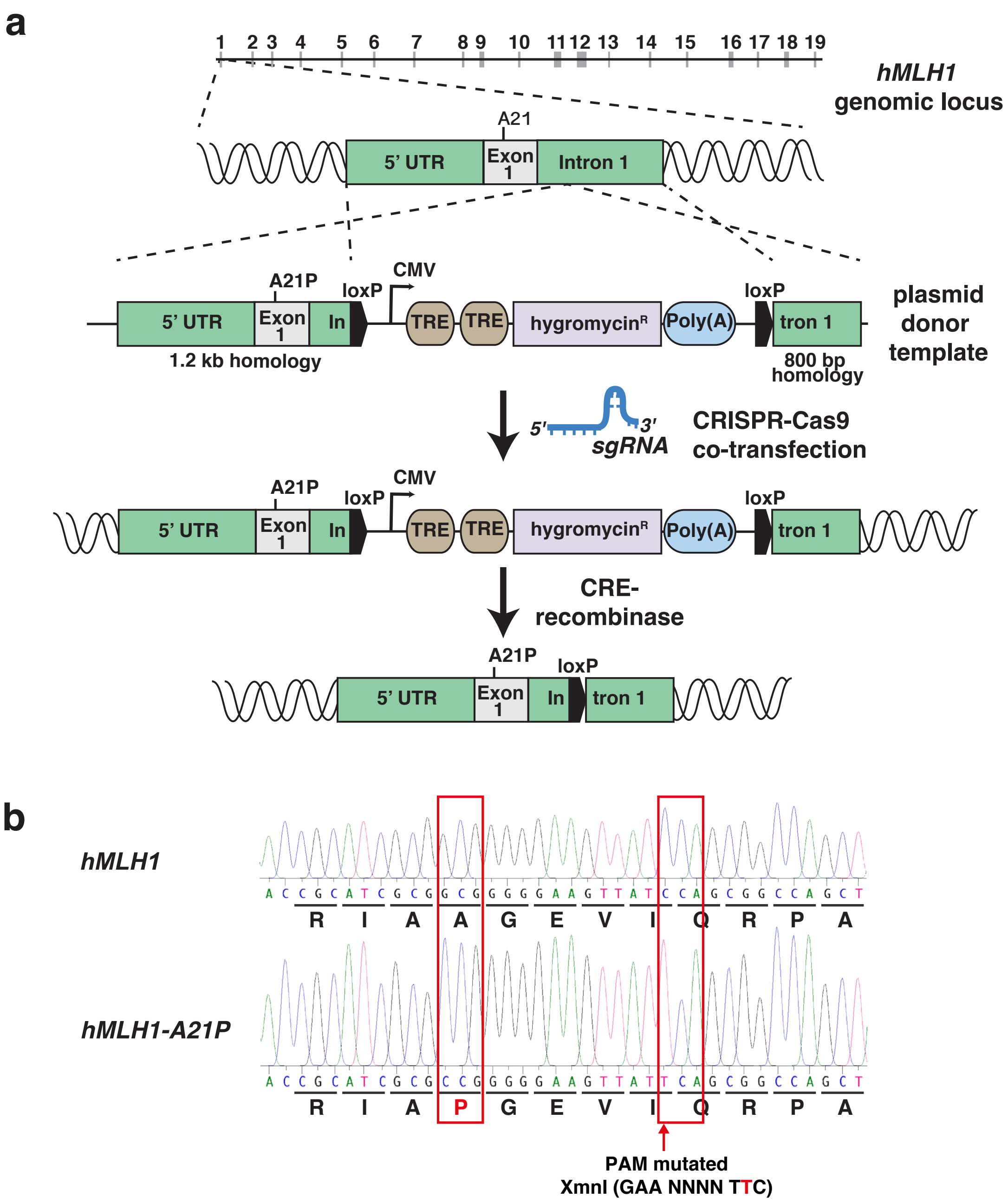

Supplementary Fig. 5. Generation of *hMLH1-A21P* HAP1 mutant cell lines. a, CRISPR-Cas9 editing strategy used to introduce the *hMLH1-A21P* point mutation in HAP1 cells. **b**, Sequencing chromatograms of a DNA fragment of the *hMLH1* gene, indicating the A21P mutation, and a mutation at the PAM motif that creates a *Xmn*I site and prevents Cas9-mediated cleavage, after successful recombination with the donor plasmid.

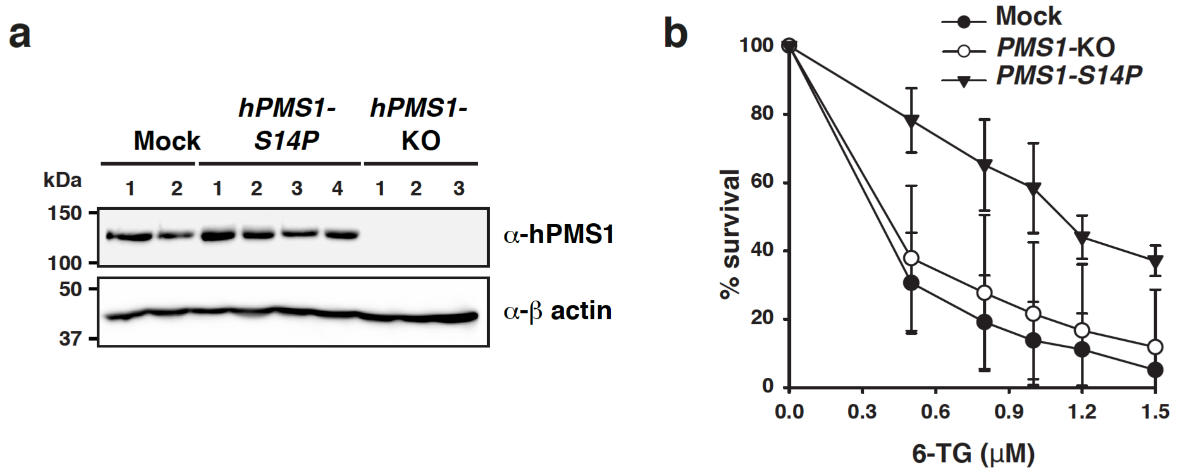

Supplementary Fig. 6. Characterization of *hPMS1-S14P* and *hPMS1*-KO HAP1 mutant cell lines. a, Total cell lysates obtained from mock-HAP1 cells, *hPMS1-S14P* and *hPMS1*-KO cells were analyzed by western blot with an antibody against human PMS1. β-actin was used as loading control. **b**, Mock-HAP1 (n=6, biologically independent clones), *hPMS1*-KO (n=5, biologically independent clones) and *hPMS1-S14P* (n=4, biologically independent clones) HAP1 cells were tested for a mutator phenotype with the *HPRT1* inactivation assay. Error bars represent standard deviation of the mean of the dataset.

Figure 3d

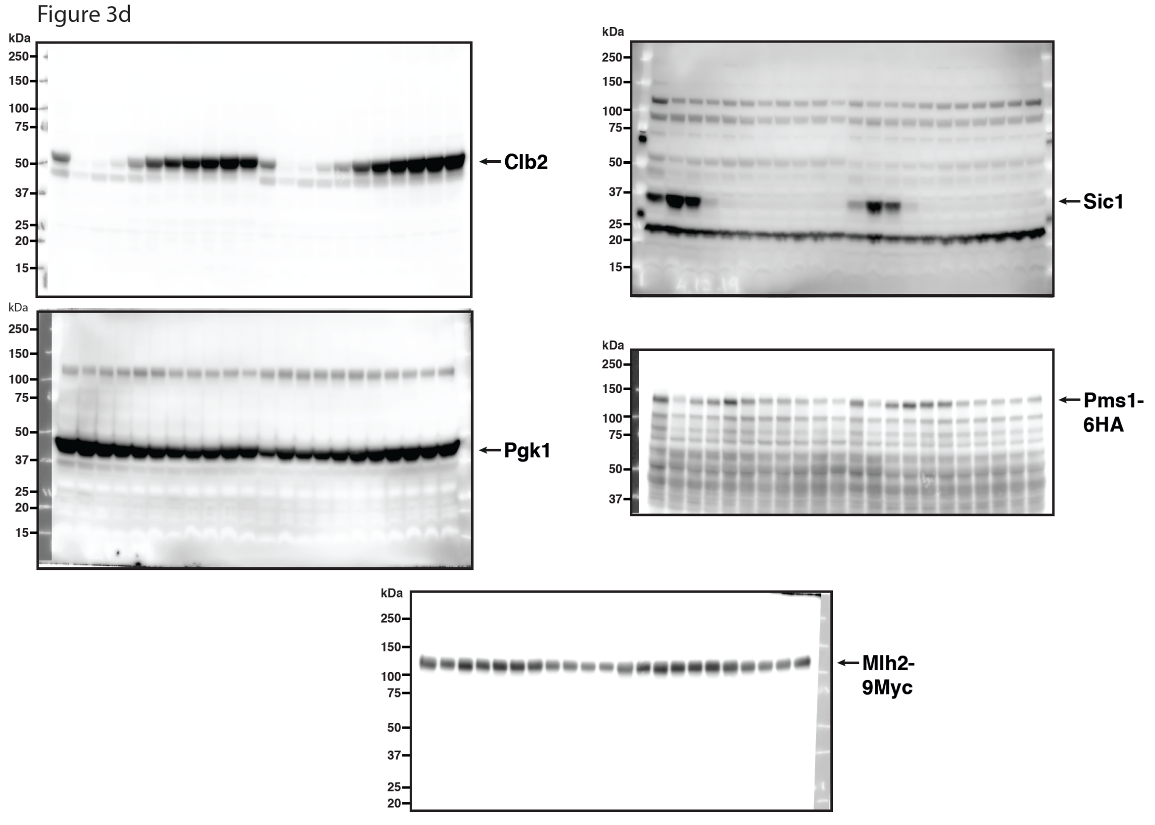

Figure 5a

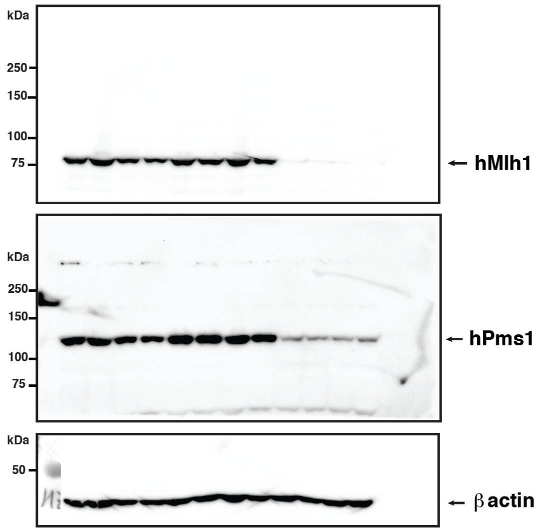

Supplementary Fig. 7. Uncropped scans of western blots Fig.3d, Fig.5a and Fig.5d.

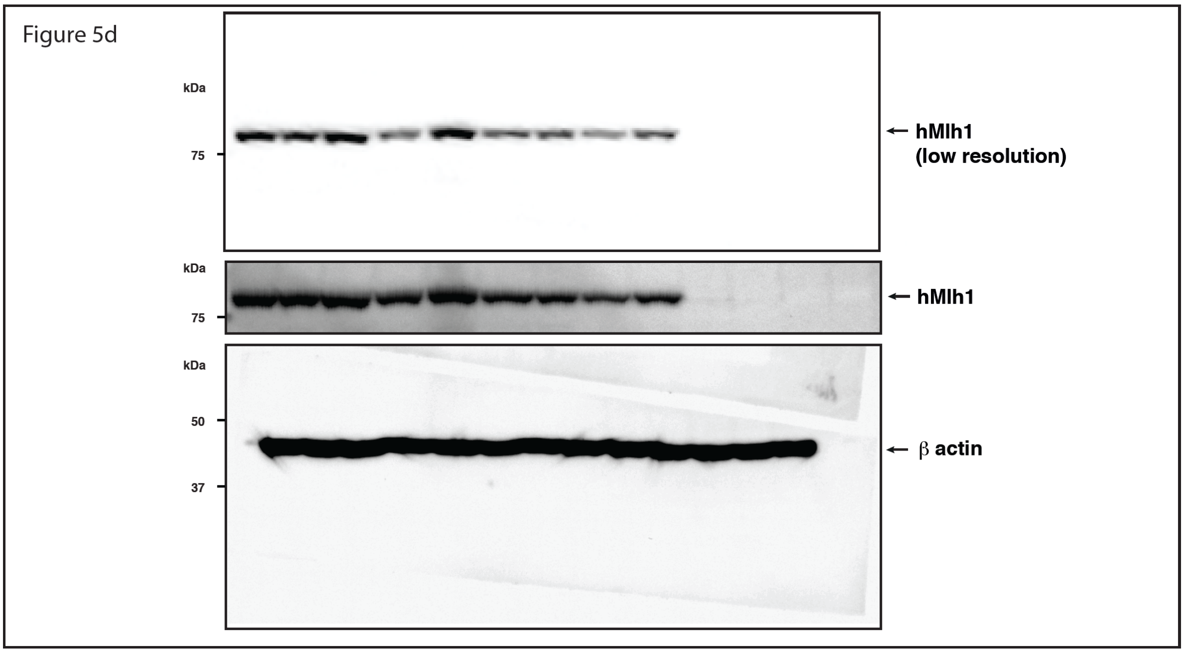

Supplementary Fig. 7 (continuation). Uncropped scans of western blots Fig.3d, Fig.5a and Fig.5d.

Supplementary Table 1. Mutation rates caused by *mlh2* mutations expressed on low copy plasmids.

	Mutation Rate (fold increase) *		
**plasmid**	**Ura^+ Lys^+**	**Ura^+ Thr^+**	**Ura^+ Can^R**
pRS316-MLH2	1.3 [1.0-1.9] x 10^{-7} (1)	1.4 [0.7-1.9] x 10^{-8} (1)	5.4 [4.6-5.9] x 10^{-7} (1)
*pRS316-mlh2-V15P***	2.3 [1.5-4.3] x 10^{-6} (18)	2.8 [2.0-3.6] x 10^{-8} (2)	4.2 [3.8-5.3] x 10^{-7} (1)
pRS316-mlh2-S16P	2.7 [2.6-4.3] x 10^{-6} (21)	4.9 [2.8-7.9] x 10^{-8} (4)	5.8 [4.4-6.8] x 10^{-7} (1)
*pRS316-mlh2-S17P***	2.7 [1.9-4.7] x 10^{-7} (2)	9.3 [7.9-14.4] x 10^{-9} (1)	5.7 [3.8-7.2] x10^{-7} (1)
pRS316-mlh2-S18P	7.3 [5.1-15.1] x 10^{-7} (6)	2.4 [1.4-3.2] x 10^{-8} (2)	4.6 [3.6-5.7] x 10^{-7} (1)
pRS316-mlh2-E99K	3.1 [1.8-4.6] x 10^{-7} (2)	1.8 [0.8-2.9] x 10^{-8} (1)	3.7 [2.5-6.6] x 10^{-7} (1)
pRS316-mlh2-P332L	6.3 [5.2-7.6] x 10^{-7} (5)	1.2 [0.8-2.1] x 10^{-8} (1)	4.4 [3.2-5.8] x 10^{-7} (1)
pRS316-mlh2-S16P-D219G	2.6 [1.9-5.3] x 10^{-5} (198)	2.1 [1.3-3.2] x 10^{-7} (15)	1.3 [1.0-1.6] x 10^{-6} (2)
pRS316-mlh2-S16P-D45N	1.4 [0.9-1.8] x 10^{-5} (106)	9.2 [7.1-16.2] x 10^{-8} (7)	7.3 [6.4-8.9] x10^{-7} (1)
pRS316-mlh2-S16P-E99K	1.7 [1.2-2.7] x 10^{-5} (129)	1.3 [0.9-1.9] x 10^{-7} (9)	8.0 [6.3-13.9] x 10^{-7} (1)
pRS316-mlh2-S16P-F177L	1.1 [0.6-1.9] x 10^{-5} (88)	8.0 [6.1-10.1] x 10^{-8} (6)	7.3 [4.9-9.1] x 10^{-7} (1)
pRS316-mlh2-S16P-D178N	4.5 [2.4-6.9] x 10^{-5} (353)	2.7 [2.0-4.1] x 10^{-7} (20)	1.1 [0.8-1.3] x 10^{-6} (2)
pRS316-mlh2-S16P-E216G	1.2 [0.7-1.4] x 10^{-5} (94)	8.2 [6.0-11.7] x 10^{-8} (6)	7.7 [5.4-9.5] x 10^{-7} (1)
pRS316-mlh2-S16P-T217A	2.0 [1.5-3.6] x 10^{-5} (154)	1.3 [0.9-1.8] x 10^{-7} (9)	9.3 [6.4-11.4] x 10^{-7} (2)

* Median rates measured with frameshift reversion assays *lys2-10A* (Ura^+ Lys^+) and *hom3-10* (Ura^+ Thr^+) or based on the inactivation of the *CAN1* gene (Ura^+ Can^R), with 95% confidence interval in square brackets and fold increase relative to WT strain in parentheses. The indicated plasmids were transformed into the WT strain (RDKY5964) and were selected in medium lacking uracil. ** Mutations were introduced by site-directed mutagenesis.

Supplementary Table 2. Mutation rate analysis in strains expressing *mlh2* dominant mutations integrated at the *MLH2* chromosomal locus.

	Mutation Rate (fold increase)*		
**Relevant genotype**	**Lys^+**	**Thr^+**	**Can^R**
WT	1.5 [0.8-2.2] x 10^{-8} (1)	2.1 [1.4-3.2] x 10^{-9} (1)	7.2 [5.7-9.0] x 10^{-8} (1)
msh2Δ	9.9 [8.1-10.8] x 10^{-5} (6771)	6.3 [5.2-12.8] x 10^{-6} (3053)	5.4 [4.4-7.2] x 10^{-6} (75)
mlh2Δ	6.0 [5.4-6.5] x 10^{-8} (4)	8.0 [4.6-11.7] x 10^{-9} (4)	8.8 [6.1-13.2] x 10^{-8} (1)
*mlh2-V15P***	6.0 [4.6-8.5] x 10^{-7} (41)	1.8 [1.2-3.5] x 10^{-8} (9)	2.0 [1.2-3.4] x 10^{-7} (3)
mlh2-S16P	9.1 [6.4-11.3] x 10^{-7} (62)	1.2 [0.9-1.7] x 10^{-8} (6)	1.0 [0.8-1.3] x 10^{-7} (1)
*mlh2-S17P***	4.1 [3.6-7.4] x 10^{-8} (3)	3.0 [2.7-5.9] x 10^{-9} (1)	1.2 [0.8-2.1] x 10^{-7} (2)
mlh2-S18P	1.5 [1.2-2.4] x 10^{-7} (10)	6.5 [5.4-10.6] x 10^{-9} (3)	1.5 [1.0-2.0] x 10^{-7} (2)
mlh2-D219G	2.4 [1.8-5.1] x 10^{-8} (2)	4.5 [1.3-6.9] x 10^{-9} (2)	1.4 [1.1-2.2] x 10^{-7} (2)
mlh2-P332L	1.2 [0.8-1.4] x 10^{-7} (8)	3.9 [2.1-6.2] x 10^{-9} (2)	1.1 [0.7-1.5] x 10^{-7} (2)
mlh2-S16P-D219G	1.1 [0.8-2.2] x 10^{-5} (737)	8.7 [6.5-15.4] x 10^{-8} (42)	1.9 [1.4-4.3] x 10^{-7} (3)
mlh2-S16P-D45N	5.6 [3.3-9.2] x 10^{-6} (381)	1.0 [0.7-1.8] x 10^{-7} (50)	1.6 [1.2-3.4] x 10^{-7} (2)
mlh2-S16P-E99K	1.5 [1.0-2.1] x 10^{-5} (1013)	2.6 [1.2-3.0] x 10^{-7} (123)	3.6 [2.4-5.7] x 10^{-7} (5)
mlh2-S16P-D178N	1.2 [0.8-2.0] x 10^{-5} (828)	2.7 [1.6-3.7] x 10^{-7} (128)	3.3 [1.7-4.9] x 10^{-7} (5)

* Median rates measured with frameshift reversion assays (*lys2-10A* (Lys^+) and *hom3-10* (Thr^+)) or based on the inactivation of the *CAN1* gene (Can^R), with 95% confidence interval in square brackets and fold increase relative to WT strain in parentheses. A strain with total loss of mismatch repair activity (*msh2Δ*) was included as reference. ** Mutations were introduced by site-directed mutagenesis (not found in our screen).

Supplementary Table 3. Mutation rates caused by *mlh2* mutations in MMR compromised strains and other relevant mutant backgrounds.

	Relevant genotype	Mutation Rate (fold increase)* Lys$^+$	Thr$^+$	Can^R
**A**	WT	1.5 [0.8-2.2] x 10^{-8} (1)	2.1 [1.4-3.2] x 10^{-9} (1)	2 [5.7-9.0] x 10^{-8} (1)
	msh2Δ	9.9 [8.1-10.8] x 10^{-5} (6771)	6.3 [5.2-12.8] x 10^{-6} (3053)	4 [4.4-7.2] x 10^{-6} (75)
	mlh2Δ	6.0 [5.4-6.5] x 10^{-8} (4)	8.0 [4.6-11.7] x 10^{-9} (4)	8.8 [6.1-13.2] x 10^{-8} (1)
	exo1Δ	1.4 [0.9-1.8] x 10^{-7} (10)	8.7 [6.1-15.0] x 10^{-9} (4)	7.4 [6.3-9.8] x 10^{-7} (10)
	pol30-K217E	3.6 [2.6-4.8] x 10^{-7} (24)	1.2 [0.9-1.8] x 10^{-8} (6)	5.7 [3.9-16.8] x 10^{-7} (8)
	pms1-A99V	2.3 [1.6-3.6] x 10^{-7} (16)	4.0 [1.0-6.6] x 10^{-8} (19)	2.2 [1.1-5.0] x 10^{-7} (3)
	mlh2-S16P	9.1 [6.4-11.3] x 10^{-7} (62)	1.2 [0.9-1.7] x 10^{-8} (6)	1.0 [0.8-1.3] x 10^{-7} (1)
	exo1Δ mlh2-S16P	8.6 [4.4-13.0] x 10^{-6} (585)	1.2 [0.9-3.3] x 10^{-7} (56)	5.7 [4.8-9.8] x 10^{-7} (8)
	mlh2-S16P pol30-K217E	3.6 [3.3-4.3] x 10^{-5} (2458)	1.5 [1.3-2.6] x 10^{-6} (717)	1.7 [1.3-2.2] x 10^{-6} (24)
	mlh2-S16P pms1-A99V	1.4 [1.0-2.1] x 10^{-5} (935)	7.0 [5.5-29.7] x 10^{-7} (338)	2.9 [2.4-4.8] x 10^{-7} (4)
	exo1Δ pol30-K217E	5.5 [4.8-7.0] x 10^{-5} (3772)	2.7 [1.6-4.2] x 10^{-6} (1317)	7.1 [3.7-10.8] x 10^{-6} (99)
	mlh2-S16P-D219G	1.1 [0.8-2.2] x 10^{-5} (737)	8.7 [6.5-15.4] x 10^{-8} (42)	1.9 [1.4-4.3] x 10^{-7} (3)
	exo1Δ mlh2-S16P-D219G	3.0 [2.3-6.3] x 10^{-5} (2042)	6.1 [4.1-9.3] x 10^{-7} (291)	1.2 [0.9-1.8] x 10^{-6} (17)
**B**	*mlh2-E29A*	1.9 [1.3-3.2] x 10^{-8} (1)	3.6 [1.4-7.7] x 10^{-9} (2)	7.2 [5.4-13.6] x 10^{-8} (1)
	mlh2-S16P-E29A	5.5 [5.3-7.6] x 10^{-8} (4)	3.3 [3.3-7.2] x 10^{-9} (2)	8.5 [6.9-16.4] x 10^{-8} (1)
	mlh2-S16P-D219G-E29A	7.6 [4.1-11.9] x 10^{-7} (52)	1.2 [0.8-2.1] x 10^{-8} (6)	1.4 [1.0-2.7] x 10^{-7} (2)
	mlh2-S16P-D219G-K294E	5.1 [4.1-5.5] x 10^{-8} (3)	2.5 [1.4-4.9] x 10^{-9} (1)	6.5 [4.4-20.0] x 10^{-8} (1)
	POL30-OE	7.6 [5.5-18.3] x 10^{-9} (1)	2.4 [1.1-4.2] x 10^{-9} (1)	7.7 [4.4-9.5] x 10^{-8} (1)
	PMS1-OE	6.8 [5.3-10.5] x 10^{-8} (5)	6.6 [4.8-8.7] x 10^{-9} (3)	9.2 [7.6-12.3] x 10^{-8} (1)
	POL30-OE mlh2-S16P-D219G	2.1 [1.6-2.6] x 10^{-6} (145)	2.7 [2.0-5.2] x 10^{-8} (13)	8.3 [6.4-10.8] x 10^{-8} (1)
	PMS1-OE mlh2-S16P-D219G	2.1 [1.4-3.4] x 10^{-7} (15)	1.5 [1.0-1.8] x 10^{-8} (7)	1.0 [0.6-1.4] x 10^{-7} (1)
**C**	*pms1-S17P*	1.9 [1.5-2.5] x 10^{-5} (1319)	5.0 [3.5-8.5] x 10^{-7} (239)	7.2 [4.0-14.4] x 10^{-7} (10)
	mlh1-A18P	1.3 [1.0-2.5] x 10^{-4} (8921)	6.3 [4.5-9.9] x 10^{-6} (3020)	4.6 [2.8-6.7] x 10^{-6} (64)

* Median rates measured with frameshift reversion assays (*lys2-10A* (Lys$^+$) and *hom3-10* (Thr$^+$)) or based on the inactivation of the *CAN1* gene (Can^R), with 95% confidence interval in square brackets and fold increase relative to WT strain in parentheses. A strain with a total loss of mismatch repair activity (*msh2Δ*) was included as reference.

Supplementary Table 4. *CAN1* mutation spectrum in *mlh2* mutant strains.

		Insertion / deletion		Base change		Complex
**Genotype**		**Mutation**	**Occurrence**	**Mutation**	**Occurrence**	**Occurrence**
**wild-type**	ΔA	A6 → A5	1 (1)	A-T → G-C	6 (7)	8 (9)
	ΔT	T6 → T5	2 (2)	G-C → A-T	18 (20)	
		T2 → T1	2 (2)	G-C → T-A	29 (32)	
	ΔC	C2 → C1	1 (1)	A-T → C-G	3 (3)	
		C1 → C0	2 (2)	A-T → T-A	7 (8)	
	+T	T6 → T7	3 (3)	C-G → G-C	6 (7)	
		T2 → T3	3 (3)			
	+G	G2 → G3	1 (1)			
			**15 (16)**		**69 (75)**	**8 (9)**
mlh2-S16P-E99K	ΔA	A6 → A5	13 (14)	A-T → G-C	3 (3)	2 (2)
		A5 → A4	2 (2)	G-C → A-T	12 (13)	
		A3 → A2	1 (1)	G-C → T-A	10 (11)	
	ΔT	T6 → T5	16 (18)	A-T → C-G	1 (1)	
		T5 → T4	3 (3)	A-T → T-A	3 (3)	
		T4 → T3	8 (9)	C-G → G-C	2 (2)	
		T3 → T2	3 (3)			
		T1 → T0	1 (1)			
	ΔG	G4 → G3	2 (2)			
		G2 → G1	1 (1)			
	+A	A6 → A7	1 (1)			
		A5 → A6	2 (2)			
		A4 → A5	1 (1)			
	+T	T6 → T7	2 (2)			
		T4 → T5	1 (1)			
			**57 (63)**		**31 (34)**	**2 (2)**
mlh2-S16P-D219G	ΔA	A6 → A5	14 (15)	A-T → G-C	2 (2)	4 (4)
		A5 → A4	2 (2)	G-C → A-T	12 (13)	
		A4 → A3	1 (1)	G-C → T-A	8 (9)	
		A2 → A1	1 (1)	A-T → C-G	0 (0)	
	ΔT	T6 → T5	19 (20)	A-T → T-A	2 (2)	
		T5 → T4	6 (6)	C-G → G-C	3 (3)	
		T4 → T3	12 (13)			
		T1 → T0	1 (1)			
	ΔG	G4 → G3	1 (1)			
		G2 → G1	1(1)			
	ΔC	C1 → C0	2 (2)			
	+T	T6 → T7	2 (2)			
			**62 (67)**		**27 (29)**	**4 (4)**

The *CAN1* mutation spectra based on DNA sequencing of individual Can^R mutants, shown as the number of clones containing the indicated mutations, and in parenthesis as the percentage relative to the total.

Supplementary Table 5. *MLH2* mutations identified in this study and homolog human MLH mutations reported in cancer-genome databases.

Sequence or motif mutated	*S. cerevisiae MLH2* mutation	Human homolog mutation	Database	Pathogenicity
	V15P	*PMS1-S13L*	TCGA, ClinVar	Likely pathogenic/VUS
		MLH1-A20T	ClinVar	VUS
S16, S17, S18 (loop L1)	*S16P*	*MLH1-A21V*	InSIGHT, ClinVar	Pathogenic
		MLH1-A21E	ClinVar	Pathogenic
		PMS2-S28P	ClinVar	VUS
	S17P	*MLH1-G22V*	InSIGHT, ClinVar	VUS
		PMS2-G29A	ClinVar	VUS
	S18P	*MLH1-E23D*	InSIGHT	VUS
		PMS2-Q30P	COSMIC	Pathogenic
		PMS2-Q30K	ClinVar	VUS
D45 (sheet β1)	*D45N*	*MLH1-Q48E*	TCGA, ClinVar	VUS
		MLH1-Q48P	ClinVar	Likely pathogenic
		PMS2-D55V	ClinVar	VUS
E99 (ATPase motif III)	*E99K*	*MLH1-E102A*	ClinVar	Likely pathogenic
		MLH1-E102D	ClinVar	Pathogenic
		MLH1-E102K	ClinVar	Likely pathogenic
		PMS2-E109K	COSMIC, ClinVar	Pathogenic/VUS
Cluster I (helix αE)	*F177L*	*PMS2-Y181F*	ClinVar	VUS
	D178N	*MLH1-G174C*	ClinVar	VUS
		PMS2-A182T	ClinVar	VUS
Cluster II (helix αF)	*E216G*	*MLH1-A210T*	COSMIC, ClinVar	Pathogenic/VUS
		PMS2-P221S	COSMIC	VUS*
		PMS2-P221L	ClinVar	VUS
	T217A	*PMS2-S222I*	ClinVar	VUS
	D219G	*MLH1-V213L*	InSight, CinVar	VUS
		PMS2-K224N	ClinVar	VUS
P332 (loop L3)	*P332L*	*MLH1-P309L*	InSIGHT, ClinVar	VUS
		PMS2-P338L	InSIGHT, ClinVar	VUS

Information obtained from databases: InSight (www.insight-group.org/variants/databases/), ClinVar (www.ncbi.nlm.nih.gov/clinvar), COSMIC (cancer.sanger.ac.uk/cosmic), TCGA (portal.gdc.cancer.gov). VUS: variant of uncertain significance. * variant without classification of pathogenicity (but found in a tumor from a melanoma patient).

Supplementary Table 6. *S. cerevisiae* strains used in this study.

Name	Relevant genotype [a]	Reference
RDKY5964	*Mata ura3-52 leu2Δ1 trp1Δ63 hom3-10 his3Δ200 lys2-10A*	1
HHY6505	RDKY5964 *msh2::HIS3*	2
RDKY7926	RDKY5964 *mlh2::kanMX4*	3
HHY6620	RDKY5964 *lig4::HIS3*	This study
HHY8030	RDKY5964 *mlh2-V15P*	This study
HHY8031	RDKY5964 *mlh2-S16P*	This study
HHY8032	RDKY5964 *mlh2-S17P*	This study
HHY8033	RDKY5964 *mlh2-S18P*	This study
HHY8034	RDKY5964 *mlh2-E29A*	This study
HHY8035	RDKY5964 *mlh2-D219G*	This study
HHY8036	RDKY5964 *mlh2-P332L*	This study
HHY6003	RDKY5964 *mlh2-S16P-D45N*	This study
HHY5999	RDKY5964 *mlh2-S16P-E99K*	This study
HHY5994	RDKY5964 *mlh2-S16P-D178N*	This study
HHY8037	RDKY5964 *mlh2-S16P-D219G*	This study
HHY8038	RDKY5964 *mlh2-S16P-E29A-D219G*	This study
HHY7986	RDKY5964 *mlh2-S16P-D219G-K294E*	This study
HHY8039	RDKY5964 *kanMX6.pGPD-POL30*	This study
HHY8040	RDKY5964 *kanMX6.pGPD-POL30 mlh2-S16P-D219G*	This study
HHY8041	RDKY5964 *natNT2.pGPD-PMS1*	This study
HHY8042	RDKY5964 *natNT2.pGPD-PMS1 mlh2-S16P-D219G*	This study
HHY1794	RDKY5964 *exo1::hphNT1*	2
HHY5256	RDKY5964 *exo1::hphNT1 mlh2-S16P*	This study
HHY8043	RDKY5964 *exo1::hphNT1 mlh2-S16P-D219G*	This study
HHY7555	RDKY5964 *pol30-K217E::LEU2*	This study
HHY7640	RDKY5964 *pol30-K217E::LEU2 mlh2-S16P*	This study
HHY6035	RDKY5964 *pol30-K217E::LEU2 exo1::hphNT1*	This study
HHY5554	RDKY5964 *pms1-A99V*	This study
HHY7692	RDKY5964 *pms1-A99V mlh2-S16P*	This study
HHY8044	RDKY5964 *pms1-S17P*	This study
HHY8045	RDKY5964 *mlh1-A18P*	This study
HHY4867	RDKY5964 *MLH2-9Myc.hphNT1*	This study
HHY8046	RDKY5964 *mlh2-V15P-9Myc.HIS3MX6*	This study
HHY8047	RDKY5964 *mlh2-S16P-9Myc.HIS3MX6*	This study
HHY8048	RDKY5964 *mlh2-S17P-9Myc.HIS3MX6*	This study
HHY8049	RDKY5964 *mlh2-S18P-9Myc.HIS3MX6*	This study
HHY8050	RDKY5964 *mlh2-P332L-9Myc.HIS3MX6*	This study
HHY8051	RDKY5964 *mlh2-E29A-9Myc.HIS3MX6*	This study
HHY8052	RDKY5964 *mlh2-S16P-D219G-9Myc.HIS3MX6*	This study
HHY8053	RDKY5964 *mlh2-S16P-E29A-D219G-9Myc.HIS3MX6*	This study
HHY8054	RDKY5964 *mlh2-S16P-D219G-K294E-9Myc.HIS3MX6*	This study
HHY8055	RDKY5964 *MLH2-9Myc.hphNT1 PMS1-6HA.natNT2*	This study
HHY8056	RDKY5964 *mlh2-S16P-D45N-9Myc.HIS3MX6 PMS1-6HA.natNT2*	This study
HHY8057	RDKY5964 *mlh2-S16P-E99K-9Myc.HIS3MX6 PMS1-6HA.natNT2*	This study
HHY8058	RDKY5964 *mlh2-S16P-D178N-9Myc.HIS3MX6 PMS1-6HA.natNT2*	This study

HHY8059	RDKY5964 *mlh2-S16P-D219G-9Myc.HIS3MX6 PMS1-6HA.natNT2*	This study
RDKY7893	RDKY5964 *MLH2-4xGFP.kanMX6*	3
HHY8060	RDKY5964 *mlh2-S16P-4xGFP.kanMX6*	This study
HHY8061	RDKY5964 *mlh2-S16P-D45N-4xGFP.kanMX6*	This study
HHY8062	RDKY5964 *mlh2-S16P-E99K-4xGFP.kanMX6*	This study
HHY8063	RDKY5964 *mlh2-S16P-D178N-4xGFP.kanMX6*	This study
HHY8064	RDKY5964 *mlh2-S16P-D219G-4xGFP.kanMX6*	This study
HHY8065	RDKY5964 *mlh2-S16P-E29A-D219G-4xGFP.kanMX6*	This study
HHY8066	RDKY5964 *mlh2-S16P-D219G-K294E-4xGFP.kanMX6*	This study
RDKY7914	RDKY5964 *MLH2-4xGFP.kanMX6 exo1::hphNT1*	3
HHY8067	RDKY5964 *mlh2-S16P-4xGFP.kanMX6 exo1::natNT2*	This study
HHY8068	RDKY5964 *mlh2-S16P-D219G-4xGFP.kanMX6 natNT2.pGPD-POL30*	This study
HHY8069	RDKY5964 *mlh2-S16P-D219G-4xGFP.kanMX6 natNT2.pGPD-PMS1*	This study
RDKY7905	RDKY5964 *MLH2-4xGFP.kanMX6 NIC96-mCherry.hphNT1*	3
RDKY7588	RDKY5964 *PMS1-4xGFP.kanMX6*	1
HHY8071	RDKY5964 *PMS1-4xGFP.kanMX6 mlh2-S16P*	This study
HHY5392	RDKY5964 *PMS1-4xGFP.kanMX6 mlh2-D219G*	This study
HHY5396	RDKY5964 *PMS1-4xGFP.kanMX6 mlh2-P332L*	This study
HHY5326	RDKY5964 *PMS1-4xGFP.kanMX6 mlh2-S16P-D219G*	This study
RDKY7600	RDKY5964 *PMS1-4xGFP.kanMX6 SIK1-mCherry.hphNT1*	1
HHY8072	RDKY5964 *PMS1-4xGFP.kanMX6 NUP49-mCherry.hphNT1*	This study
HHY8073	RDKY5964 *PMS1-4xGFP.kanMX6 mlh2-S16P-D219G NUP49-mCherry.hphNT1*	This study
AH109	*MATa, trp1-901, leu2-3, 112, ura3-52, his3-200, gal4Δ, gal80Δ, LYS2::GAL1*$_{UAS}$-*GAL1*$_{TATA}$-*HIS3, GAL2*$_{UAS}$-*GAL2*$_{TATA}$-*ADE2, URA3::MEL1*$_{UAS}$-*MEL1*$_{TATA}$-*lacZ*	Clontech

[a] All strains derived from S288c with exception of AH109 that was used exclusively for Y2H analysis. The genotype corresponds to the listed strain with the indicated modifications.

Supplementary Table 7. Plasmids used in this study.

name	relevant genotype	base substitution(s)	reference
pRS316	*amp*^{r} *CEN6 ARSH4 URA3*	backbone	4
pHHB98	*pRS316-MLH2* (*amp*^{r} *CEN6 ARSH4 URA3)*	WT-*MLH2*	This study
pHHB218	*pRS316-mlh2-V15P* (*amp*^{r} *CEN6 ARSH4 URA3)*	c.43G>C, c.44T>C	This study
pHHB221	*pRS316-mlh2-S16P* (*amp*^{r} *CEN6 ARSH4 URA3)*	c.46T>C	This study
pHHB216	*pRS316-mlh2-S17P* (*amp*^{r} *CEN6 ARSH4 URA3)*	c.49A>C, c.50G>C	This study
pHHB217	*pRS316-mlh2-S18P* (*amp*^{r} *CEN6 ARSH4 URA3)*	c.52T>C	This study
pHHB410	*pRS316-mlh2-E99K* (*amp*^{r} *CEN6 ARSH4 URA3)*	c.295G>A	This study
pHHB129	*pRS316-mlh2-P332L* (*amp*^{r} *CEN6 ARSH4 URA3)*	c.995C>T	This study
pHHB399	*pRS316-mlh2-S16P-D45N* (*amp*^{r} *CEN6 ARSH4 URA3)*	c.46T>C, c.133G>A	This study
pHHB411	*pRS316-mlh2-S16P-E99K* (*amp*^{r} *CEN6 ARSH4 URA3)*	c.46T>C, c.295G>A	This study
pHHB418	*pRS316-mlh2-S16P-F177L* (*amp*^{r} *CEN6 ARSH4 URA3)*	c.46T>C, c.529T>C	This study
pHHB398	*pRS316-mlh2-S16P-D178N* (*amp*^{r} *CEN6 ARSH4 URA3)*	c.46T>C, c.532G>A	This study
pHHB400	*pRS316-mlh2-S16P-E216G* (*amp*^{r} *CEN6 ARSH4 URA3)*	c.46T>C, c.647A>G	This study
pHHB408	*pRS316-mlh2-S16P-T217A* (*amp*^{r} *CEN6 ARSH4 URA3)*	c.46T>C, c.649A>G	This study
pHHB143	*pRS316-mlh2-S16P-D219G* (*amp*^{r} *CEN6 ARSH4 URA3)*	c.46T>C, c.656A>G	This study
pRS306	*amp*^{r} *URA3* integrative vector	backbone	4
pHHB270	*pRS306-mlh1-A18P* (*amp*^{r} *URA3)*	c.52G>C	This study
pHHB157	*pRS306-MLH2* (*amp*^{r} *URA3)*	WT-*MLH2*	This study
pHHB225	*pRS306-mlh2-V15P* (*amp*^{r} *URA3)*	c.43G>C, c.44T>C	This study
pHHB170	*pRS306-mlh2-S16P* (*amp*^{r} *URA3)*	c.46T>C	This study
pHHB223	*pRS306-mlh2-S17P* (*amp*^{r} *URA3)*	c.49A>C, c.50G>C	This study
pHHB224	*pRS306-mlh2-S18P* (*amp*^{r} *URA3)*	c.52T>C	This study
pHHB231	*pRS306-mlh2-E29A* (*amp*^{r} *URA3)*	c.86A>C	This study
pHHB180	*pRS306-mlh2-D219G* (*amp*^{r} *URA3)*	c.656A>G	This study
pHHB222	*pRS306-mlh2-P332L* (*amp*^{r} *URA3)*	c.995C>T	This study
pHHB298	*pRS306-mlh2-S16P-E29A* (*amp*^{r} *URA3)*	c.46T>C, c.86A>C	This study
pHHB433	*pRS306-mlh2-S16P-D45N* (*amp*^{r} *URA3)*	c.46T>C, c.133G>A	This study
pHHB434	*pRS306-mlh2-S16P-E99K* (*amp*^{r} *URA3)*	c.46T>C, c.295G>A	This study
pHHB435	*pRS306-mlh2-S16P-D178N* (*amp*^{r} *URA3)*	c.46T>C, c.532G>A	This study
pHHB226	*pRS306-mlh2-S16P-D219G* (*amp*^{r} *URA3)*	c.46T>C, c.656A>G	This study
pHHB272	*pRS306-mlh2-S16P-D219G-E29A* (*amp*^{r} *URA3)*	c.46T>C, c.656A>G, c.86A>C	This study
pHHB593	*pRS306-mlh2-S16P-D219G-K294E* (*amp*^{r} *URA3)*	c.46T>C, c.656A>G, c.880A>G	This study
pHHB240	*pRS306-pms1-S17P* (*amp*^{r} *URA3)*	c.49T>C, c.51T>A	This study
pHHB252	*pol30-K217E::LEU2 (amp*^{r} *CEN6 ARSH4 TRP1)*	c.649A>G	This study
pGBKT7	*GAL4 DBD* yeast two-hybrid bait vector *(kan*^{r} *2μ TRP1*)	backbone	Clontech
pGADT7	*GAL4 AD* yeast two-hybrid prey vector (*amp*^{r} *2μ LEU2*)	backbone	Clontech
pHHB309	*pGBKT7-PMS1* (*kan*^{r} *2μ TRP1*)	WT-*PMS1*	This study
pHHB307	*pGBKT7-MLH2 (kan*^{r} *2μ TRP1)*	WT-*MLH2*	This study
pHHB341	*pGBKT7-mlh2-S16P* (*kan*^{r} *2μ TRP1)*	c.46T>C	This study
pHHB308	*pGBKT7-mlh2-S16P-D219G (kan*^{r} 2*μ TRP1)*	c.46T>C, c.656A>G	This study
pHHB455	*pGBKT7-mlh2-S16P-E99K (kan*^{r} *2μ TRP1)*	c.46T>C, c.295G>A	This study
pHHB456	*pGBKT7-mlh2-S16P-D178N (kan*^{r} 2*μ TRP1)*	c.46T>C, c.532G>A	This study
pHHB1295	*pGBKT7-mlh2-S16P-D219G (kan*^{r} 2*μ TRP1)*	c.46T>C, c.656A>G, c.86A>C	This study
pHHB1296	*pGBKT7-mlh2-S16P-D219G (kan*^{r} 2*μ TRP1)*	c.46T>C, c.656A>G, c.880A>G	This study

pHHB311	*pGADT7-MLH1 (amp^r 2μ LEU2)*	WT-*MLH1*	This study
pLentiCRISPR	*sgRNA, Cas9, puro^r*	backbone	Addgene
pcDNA5 FRT/TO	*pCMV amp^r 2xtetO$_2$ FRT* site, hygromycin (without ATG)	backbone	Invitrogen
pHHB761	*pLentiCRISPR-puro-sgRNA-GFP*	sgRNA-GFP	This study
pHHB586	*pLentiCRISPR-puro-sgRNA-hMLH1*	sgRNA-human *hMLH1*	This study
pHHB484	*pLentiCRISPR-puro-sgRNA-hPMS1*	sgRNA-human *hPMS1*	This study
pHHB487	*pLentiCRISPR-puro-sgRNA-hPMS1*	sgRNA-human *hPMS1*	This study
pHHB690	pcDNA5 FRT/TO-*hygro(loxP)-hMLH1-A21P*	*hMLH1-A21P*, PAM	This study
pHHB730	pcDNA5 FRT/TO-*hygro(loxP)-hPMS1-S14P*	*hPMS1-S14P*, PAM, *Psi*I site	This study

Supplementary references

1 Hombauer, H., Campbell, C. S., Smith, C. E., Desai, A. & Kolodner, R. D. Visualization of eukaryotic DNA mismatch repair reveals distinct recognition and repair intermediates. *Cell* **147**, 1040-1053, doi:10.1016/j.cell.2011.10.025 (2011).

2 Schmidt, T. T. *et al.* Alterations in cellular metabolism triggered by URA7 or GLN3 inactivation cause imbalanced dNTP pools and increased mutagenesis. *Proc Natl Acad Sci U S A* **114**, E4442-E4451, doi:10.1073/pnas.1618714114 (2017).

3 Campbell, C. S. *et al.* Mlh2 is an accessory factor for DNA mismatch repair in Saccharomyces cerevisiae. *PLoS genetics* **10**, e1004327, doi:10.1371/journal.pgen.1004327 (2014).

4 Sikorski, R. S. & Hieter, P. A system of shuttle vectors and yeast host strains designed for efficient manipulation of DNA in Saccharomyces cerevisiae. *Genetics* **122**, 19-27 (1989).
