# Supplementary material for: Identification of MLH2/hPMS1 dominant mutations that prevent DNA mismatch repair function
Source: Commun Biol. 2020 Dec 10;3:751. doi: 10.1038/s42003-020-01481-4 (PMC7730388; doi:10.1038/s42003-020-01481-4)
Supplement: Supplementary file 2 — Description of Additional Supplementary File [file 42003_2020_1481_MOESM2_ESM.pdf]

**Description of additional supplementary file**

**File name:** Supplementary Data 1

**Description:** Source data for the main and supplementary figures
