# Supplementary material for: Identification of MLH2/hPMS1 dominant mutations that prevent DNA mismatch repair function
Source: Commun Biol. 2020 Dec 10;3:751. doi: 10.1038/s42003-020-01481-4 (PMC7730388; doi:10.1038/s42003-020-01481-4)
Supplement: Supplementary file 4 — Reporting Summary [file 42003_2020_1481_MOESM4_ESM.pdf]

nature research

Corresponding author(s): Hans Hombauer

Last updated by author(s): Oct 17, 2020

Reporting Summary

Nature Research wishes to improve the reproducibility of the work that we publish. This form provides structure for consistency and transparency in reporting. For further information on Nature Research policies, see our Editorial Policies and the Editorial Policy Checklist.

Statistics

For all statistical analyses, confirm that the following items are present in the figure legend, table legend, main text, or Methods section.

n/a	Confirmed	
[ ]	[x]	The exact sample size (n) for each experimental group/condition, given as a discrete number and unit of measurement
[ ]	[x]	A statement on whether measurements were taken from distinct samples or whether the same sample was measured repeatedly
[ ]	[x]	The statistical test(s) used AND whether they are one- or two-sided<br>*Only common tests should be described solely by name; describe more complex techniques in the Methods section.*
[x]	[ ]	A description of all covariates tested
[x]	[ ]	A description of any assumptions or corrections, such as tests of normality and adjustment for multiple comparisons
[ ]	[x]	A full description of the statistical parameters including central tendency (e.g. means) or other basic estimates (e.g. regression coefficient) AND variation (e.g. standard deviation) or associated estimates of uncertainty (e.g. confidence intervals)
[x]	[ ]	For null hypothesis testing, the test statistic (e.g. F, t, r) with confidence intervals, effect sizes, degrees of freedom and P value noted<br>*Give P values as exact values whenever suitable.*
[x]	[ ]	For Bayesian analysis, information on the choice of priors and Markov chain Monte Carlo settings
[x]	[ ]	For hierarchical and complex designs, identification of the appropriate level for tests and full reporting of outcomes
[x]	[ ]	Estimates of effect sizes (e.g. Cohen's d, Pearson's r), indicating how they were calculated

Our web collection on statistics for biologists contains articles on many of the points above.

Software and code

Policy information about availability of computer code

Data collection	Lasergene 15.1 (DNASTAR)<br>DeltaVision (Applied Precision, Inc)<br>Leica SP5 confocal<br>BD FACS CantoII
Data analysis	SigmaPlot version 10<br>SoftWoRx deconvolution<br>ImageJ 2.0.0.<br>Leica application suite (LAS AF)<br>Lasergene 15.1, Protean 3D (DNASTAR)<br>BD FACS DIVA version 8

For manuscripts utilizing custom algorithms or software that are central to the research but not yet described in published literature, software must be made available to editors and reviewers. We strongly encourage code deposition in a community repository (e.g. GitHub). See the Nature Research guidelines for submitting code & software for further information.

Data

Policy information about availability of data

All manuscripts must include a data availability statement. This statement should provide the following information, where applicable:

- Accession codes, unique identifiers, or web links for publicly available datasets
- A list of figures that have associated raw data
- A description of any restrictions on data availability

All data generated or analyzed during this study are included in this published article (and its supplementary information files).

Field-specific reporting

Please select the one below that is the best fit for your research. If you are not sure, read the appropriate sections before making your selection.

[x] Life sciences [ ] Behavioural & social sciences [ ] Ecological, evolutionary & environmental sciences

For a reference copy of the document with all sections, see nature.com/documents/nr-reporting-summary-flat.pdf

Life sciences study design

All studies must disclose on these points even when the disclosure is negative.

Sample size	No statistical methods were used to predetermine sample size. Sample sizes were determined by magnitude and consistency of measurable differences.
Data exclusions	No data were excluded.
Replication	Experiments are representative of 2-3 replicates as described in the figure legends/methods. Attempts at replication were successful. Mutation rate analysis was calculated using two independent biological isolates and a total of at least 14 independent cultures.
Randomization	at least two independent biological isolates were analyzed per experiment and gave similar results.
Blinding	Investigators were not blinded during group allocation and data analysis.

Reporting for specific materials, systems and methods

We require information from authors about some types of materials, experimental systems and methods used in many studies. Here, indicate whether each material, system or method listed is relevant to your study. If you are not sure if a list item applies to your research, read the appropriate section before selecting a response.

Materials & experimental systems

n/a	Involved in the study	
[ ]	[x]	Antibodies
[ ]	[x]	Eukaryotic cell lines
[x]	[ ]	Palaeontology and archaeology
[ ]	[x]	Animals and other organisms
[x]	[ ]	Human research participants
[x]	[ ]	Clinical data
[x]	[ ]	Dual use research of concern

Methods

n/a	Involved in the study	
[x]	[ ]	ChIP-seq
[x]	[ ]	Flow cytometry
[x]	[ ]	MRI-based neuroimaging

Antibodies

Antibodies used	Primary antibodies (company, clone number, dilution): mouse anti-MYC (Millipore, 4A6, 1:1,000), rat anti-HA (Roche, 3F10, 1:5,000), rabbit anti-Clb2 (Santa Cruz, sc-9071, 1:1,000), mouse anti-Pgk1 (Invitrogen, 22C5D8, 1:20,000), guinea pig anti-Sic1 (Schmidt et al., 2019, 1:10,000), mouse anti-hMLH1, (BD-Pharmingen, BD-551091, 1:1,000), rabbit anti-hPMS1 (Santa Cruz, sc-615, 1:1,000) and mouse anti-actin (Sigma, A2228, 1:5,000).<br>Secondary antibodies: rabbit anti-guinea pig IgG conjugated to horseradish peroxidase (HRP) (Bethyl Laboratories, A60211P, 1:10,000), sheep anti-mouse IgG-HRP (GE Healthcare, NA9310, 1:10,000), donkey anti-rabbit-HRP (GE Healthcare, NA934, 1:10,000), goat anti-rat IgG-HRP (Calbiochem, 401416, 1:10,000).
Validation	Antibodies against yeast proteins (or tags) were validated by western blotting using control yeast cell lysates of strains presenting or not the epitope recognized by the antibody.

Eukaryotic cell lines

Policy information about cell lines

Cell line source(s)	HAP1 cells were bought from Haplogen (Horizon Discovery)
Authentication	HAP1 cells were not authenticated
Mycoplasma contamination	HAP1 cell lines and derivatives were tested negative for mycoplasma contamination
Commonly misidentified lines (See ICLAC register)	none

Animals and other organisms

Policy information about studies involving animals; ARRIVE guidelines recommended for reporting animal research

Laboratory animals	this study did not involved laboratory animals
Wild animals	This study did not use wild animals.
Field-collected samples	This study did not use field-collected samples
Ethics oversight	n/a

Note that full information on the approval of the study protocol must also be provided in the manuscript.
